# Guideline-Directed Medical Therapy for Diabetic Kidney Disease: Advances in Primary and Secondary Prevention

**DOI:** 10.7759/cureus.101867

**Published:** 2026-01-19

**Authors:** Sidhant Verma, Susant Katwal, Harshika Reddy Akula, Alfiya Inamdar, Sarah S Kundukulam, Mariyam Khan, Ahmad Bin Abdul Qayyum Satti, Shweta Menon, Manju Rai

**Affiliations:** 1 Internal Medicine, Soochow University Medical College, Suzhou, CHN; 2 General Medicine, Rakhyut Hospital, Ministry of Health, Dhofar, OMN; 3 Internal Medicine, Gandhi Medical College, Secunderabad, IND; 4 Internal Medicine, Kanachur Institute of Medical Sciences, Mangalore, IND; 5 Acute Medicine and General Medicine, Medical University of Varna, Varna, BGR; 6 Internal Medicine, Charles University, Prague, CZE; 7 Internal Medicine, King Edward Medical University, Lahore, PAK; 8 General Medicine, Northern General Hospital, Sheffield, GBR; 9 Biotechnology, Shri Venkateshwara University, Gajraula, IND

**Keywords:** diabetic kidney disease, glp-1 receptor agonists, guideline-directed medical therapy, mineralocorticoid receptor antagonists, renin-angiotensin system blockade, sglt2 inhibitors

## Abstract

With the global rise in diabetes prevalence, the burden of diabetic kidney disease (DKD) is projected to escalate substantially, contributing to increased morbidity, mortality, and healthcare costs. This narrative review aims to synthesize contemporary evidence on guideline-directed medical therapy (GDMT) for DKD, with a specific focus on primary prevention, secondary prevention, and real-world implementation of evidence-based pharmacologic and non-pharmacologic strategies to mitigate renal and cardiovascular risk. A comprehensive literature review (published 2012-2025) was conducted, integrating international clinical guidelines, landmark randomized controlled trials, meta-analyses, and implementation studies addressing the prevention and management of DKD. Recent advances in GDMT have reshaped the therapeutic landscape of DKD. Lifestyle interventions - including dietary optimization, regular physical activity, and smoking cessation - demonstrate meaningful renoprotective and cardiometabolic benefits. Pharmacologic therapies, such as renin-angiotensin system (RAS) blockade, sodium-glucose co-transporter 2 (SGLT2) inhibitors, nonsteroidal mineralocorticoid receptor antagonists (MRAs), and glucagon-like peptide-1 receptor agonists (GLP-1 RAs), have shown robust efficacy in reducing albuminuria, slowing estimated glomerular filtration rate (eGFR) decline, and lowering cardiovascular events. Landmark trials report relative risk reductions of approximately 30-40% in kidney disease progression with SGLT2 inhibitors and 18-23% reductions in renal and cardiovascular composite outcomes with finerenone and GLP-1 RAs. Despite strong guideline endorsement, real-world uptake of GDMT remains suboptimal, particularly in low-resource healthcare settings. Early detection through routine eGFR and urine albumin-to-creatinine ratio screening, combined with timely initiation and sustained implementation of GDMT, offers the most effective strategy to alter the natural history of DKD. Integrating lifestyle modification with optimized pharmacotherapy is essential to reducing long-term renal and cardiovascular complications.

## Introduction and background

Diabetic kidney disease (DKD) remains one of the most common and devastating microvascular complications of diabetes mellitus, driving progression to end-stage kidney disease (ESKD) and markedly increasing cardiovascular morbidity and mortality. Despite the availability of effective therapies and well-established clinical guidelines, outcomes in DKD remain suboptimal, largely due to delayed diagnosis, fragmented care, and underutilization of guideline-directed medical therapy (GDMT) [1]. This persistent gap between evidence and practice underscores the need for a clinically focused synthesis of contemporary DKD management strategies.

Globally, DKD affects approximately 30-40% of individuals with diabetes, contributing substantially to the rising burden of chronic kidney disease and associated healthcare costs [2]. The prevalence of DKD continues to increase in parallel with diabetes, particularly in low- and middle-income countries, where access to early screening and multidisciplinary care remains limited. While epidemiological trends highlight the scale of the problem, the more pressing challenge lies in translating this knowledge into effective prevention and treatment strategies.

From a clinical perspective, DKD management has evolved beyond glycemic and blood pressure control to include multiple disease-modifying therapies with proven renal and cardiovascular benefits. Although randomized clinical trials have consistently demonstrated substantial reno- and cardioprotective benefits of GDMT, translation of these benefits to real-world patients, particularly those with advanced chronic kidney disease, frailty, and multiple comorbidities, remains variable [2]. This gap reflects restrictive trial eligibility criteria, treatment tolerability issues, polypharmacy, and health system-level barriers and is further compounded by inconsistent real-world implementation of key GDMT components, including renin-angiotensin system (RAS) blockade, sodium-glucose co-transporter 2 (SGLT2) inhibitors, nonsteroidal mineralocorticoid receptor antagonists (MRAs), and glucagon-like peptide-1 receptor agonists (GLP-1 RAs) [3]. Barriers include therapeutic inertia, safety concerns, cost, limited specialist access, and lack of coordinated care pathways, resulting in missed opportunities for early intervention and risk reduction [4].

While several reviews have examined individual pharmacologic classes or specific outcomes in DKD, there remains a need for an integrative, practice-oriented synthesis that links prevention, evidence-based management, and real-world implementation of GDMT across the DKD continuum. This narrative review, therefore, aims to (1) synthesize the latest evidence on GDMT for DKD, (2) compare key international guidelines, and (3) analyze the barriers to effective treatment. By providing this integrated overview, we seek to equip clinicians with a practical framework to alter the natural history of this costly and devastating disease.

## Review

Methodology

This study was designed as a narrative review to allow a broad, clinically oriented synthesis of evolving evidence on GDMT for DKD, including prevention, management, and real-world implementation. Given the heterogeneity of study designs, patient populations, and outcomes across the DKD literature, a narrative approach was considered most appropriate to contextualize evidence, highlight clinical applicability, and address implementation gaps rather than perform quantitative pooling. A comprehensive literature search was performed across major scientific databases, including PubMed/MEDLINE, Scopus, Web of Science, and Google Scholar. The literature search timeframe (January 2012 to August 2025) was selected to capture modern, practice-changing evidence, including pivotal randomized controlled trials and guideline updates that have reshaped contemporary DKD management. The search strategy combined Medical Subject Headings (MeSH) and free-text terms, such as "diabetic kidney disease", "diabetic nephropathy", "chronic kidney disease", "guideline-directed medical therapy", "SGLT2 inhibitors", "GLP-1 receptor agonists", "mineralocorticoid receptor antagonists", "renin-angiotensin system blockade", "hypertension guidelines", and "DKD prevention". Additional articles were identified by manually screening reference lists of relevant reviews, guidelines, and pivotal clinical trials.

Eligible sources included randomized controlled trials, observational cohort studies, meta-analyses, systematic reviews, and clinical practice guidelines published in peer-reviewed journals. Studies were included if they addressed the epidemiology, pathophysiology, prevention, progression, or pharmacologic and non-pharmacologic management of DKD. Evidence pertaining to special populations, including individuals with type 1 diabetes (T1D), advanced chronic kidney disease, or high cardiovascular risk, was also considered to enhance the generalizability of therapeutic insights.

**Figure 1:**
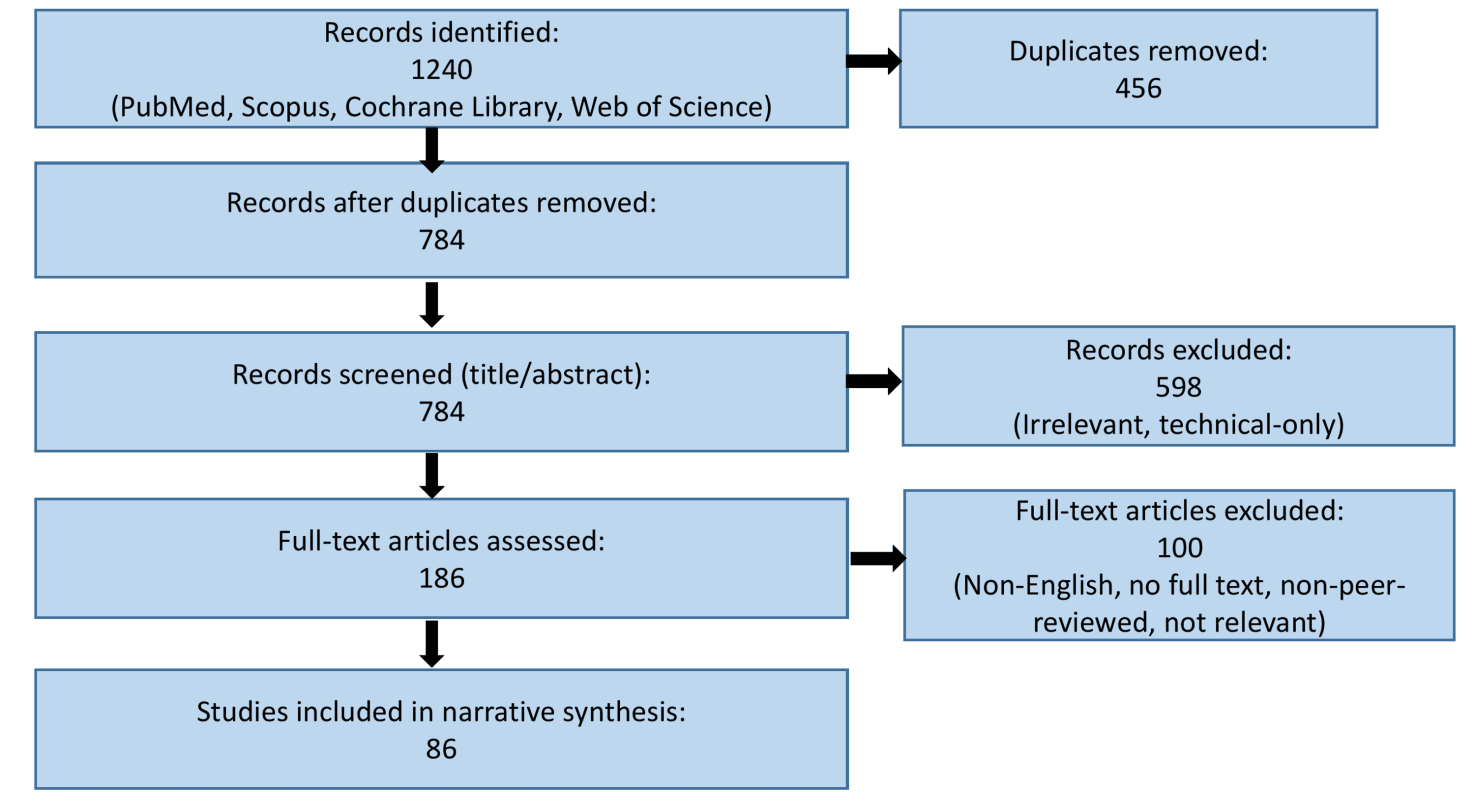
Flow diagram illustrating the literature identification and study selection process for this narrative review.

Study selection involved an initial screening of titles and abstracts, followed by a full-text review of potentially relevant articles. Exclusion criteria included lack of relevance to DKD management, pediatric populations, non-English publications, and studies without clearly defined clinical outcomes. As this review was narrative in nature, formal risk-of-bias or quality assessment tools were not applied; instead, evidence was synthesized qualitatively, with priority given to recent clinical guidelines, landmark randomized controlled trials, high-quality meta-analyses, and large observational studies with robust outcome reporting. The overall study identification and selection process is summarized in the following flow diagram (Figure 1).

Pathophysiology of DKD

DKD is a complex, multifactorial condition characterized by progressive renal functional decline arising from sustained metabolic and hemodynamic disturbances associated with diabetes. Its pathogenesis reflects the convergence of glomerular hyperfiltration, oxidative stress, inflammation, and fibrosis, ultimately leading to irreversible structural damage to the renal parenchyma.

**Figure 2:**
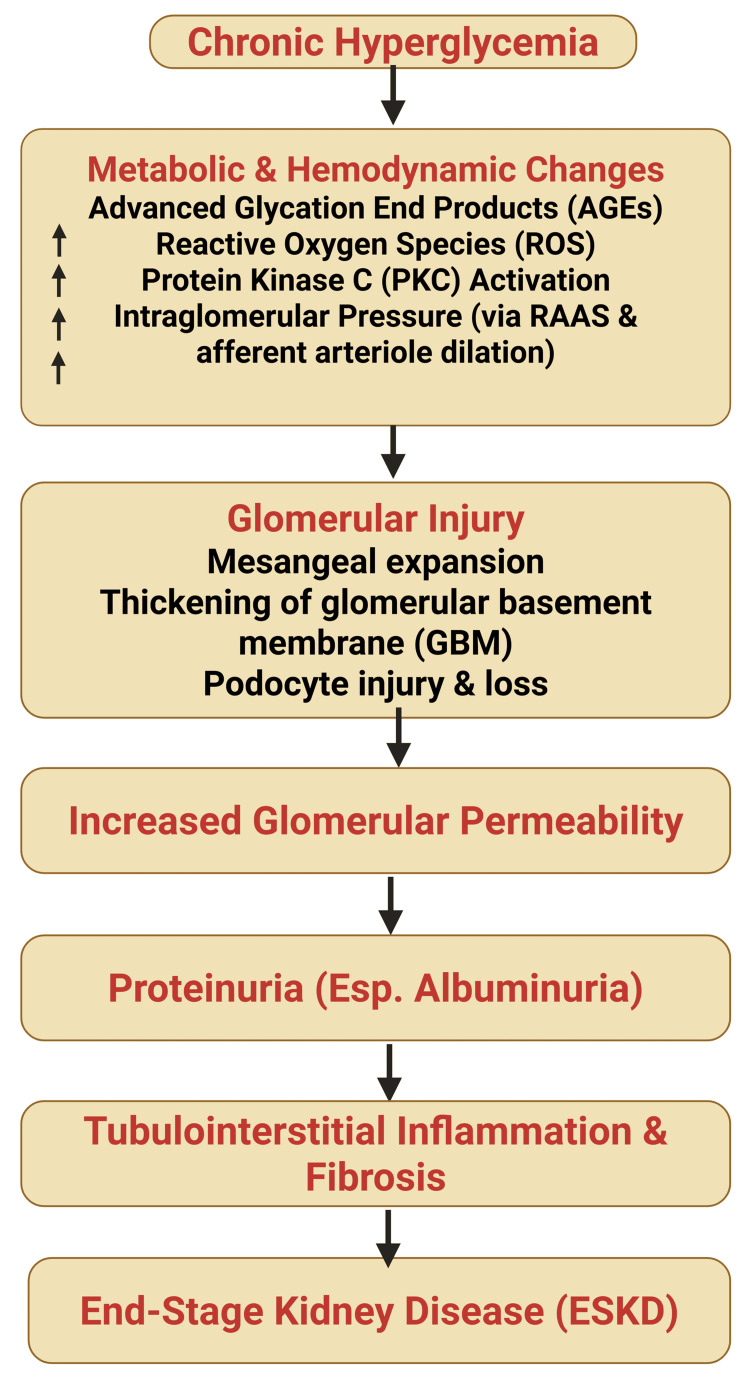
Pathophysiological cascade of diabetic kidney disease (DKD). Chronic hyperglycemia initiates a series of metabolic and hemodynamic alterations—including the formation of advanced glycation end products (AGEs), increased oxidative stress, activation of protein kinase C (PKC), and intraglomerular hypertension through renin-angiotensin-aldosterone system (RAAS) activation. These processes collectively lead to glomerular injury characterized by mesangial expansion, thickening of the glomerular basement membrane (GBM), and podocyte loss. Progressive glomerular damage increases permeability, resulting in proteinuria—especially albuminuria—which subsequently drives tubulointerstitial inflammation, fibrosis, and ultimately progression to end-stage kidney disease (ESKD). Image credit: Susant Katwal

One of the earliest pathophysiological changes in DKD is glomerular hyperfiltration, driven by afferent arteriolar vasodilation and increased intraglomerular pressure (Figure 2). Persistent hyperfiltration induces glomerular hypertrophy and mechanical stress, progressively impairing the integrity of the filtration barrier [5]. Structural alterations include glomerular basement membrane thickening, resulting from excess extracellular matrix (ECM) deposition, and podocyte loss, a key determinant of proteinuria and glomerulosclerosis [6]. Concurrent mesangial expansion, mediated by cellular proliferation and ECM accumulation, further disrupts glomerular architecture and filtration efficiency [7].

Metabolic stress plays a central role in amplifying renal injury. Chronic hyperglycemia promotes mitochondrial dysfunction and oxidative stress, leading to excessive generation of reactive oxygen species (ROS) that damage cellular lipids, proteins, and DNA [8]. This oxidative environment activates inflammatory signaling pathways, particularly nuclear factor kappa B (NF-κB), resulting in increased expression of pro-inflammatory mediators such as tumor necrosis factor-α, interleukin-6, and monocyte chemoattractant protein-1 [8]. Sustained inflammation drives immune cell infiltration into the renal interstitium, perpetuating tissue injury and accelerating disease progression [9]. These pathways provide a mechanistic rationale for therapeutic strategies targeting hemodynamic stress, inflammation, and metabolic dysregulation.

As DKD advances, fibrosis emerges as the dominant pathological process. Transforming growth factor-β (TGF-β) acts as a central profibrotic mediator by stimulating ECM synthesis and suppressing matrix degradation. Progressive tubulointerstitial fibrosis, characterized by fibroblast activation, tubular atrophy, and capillary rarefaction, culminates in irreversible renal scarring and loss of functional nephron mass [10]. Antifibrotic pathways targeted by emerging therapies, including nonsteroidal MRAs, are closely linked to these mechanisms.

Several clinical factors influence the susceptibility and progression of DKD. Poor glycemic control, reflected by elevated glycated hemoglobin levels, accelerates oxidative and inflammatory injury [11]. Hypertension increases intraglomerular pressure and shear stress, compounding structural damage [12], while dyslipidemia contributes to mesangial expansion and lipid deposition within renal tissues [13]. Genetic susceptibility also modulates disease risk, with variants in genes related to glucose metabolism, inflammation, and ECM regulation - including ACE, TGF-β1, and ELMO1 - being associated with DKD development [14]. However, while these genetic associations enhance understanding of disease biology, their current clinical utility remains limited, and their greatest relevance lies in informing future biomarker discovery and precision medicine approaches.

Clinically, DKD progresses through recognizable stages, with albuminuria representing the earliest detectable manifestation. Microalbuminuria (30-300 mg/day) often precedes macroalbuminuria (>300 mg/day), reflecting progressive glomerular barrier dysfunction [15]. This evolution is accompanied by a gradual decline in estimated eGFR, indicative of cumulative nephron loss [16]. Advanced disease is characterized by persistent proteinuria, sustained eGFR reduction (<60 mL/min/1.73 m²), and eventual progression to ESKD [17].

In summary, DKD pathophysiology reflects the interplay of metabolic stress, glomerular hyperfiltration, inflammation, and fibrosis, leading to progressive and often irreversible renal damage. Importantly, these mechanisms underpin the therapeutic rationale for contemporary guideline-directed medical therapies and emerging targeted interventions. A mechanistic understanding of DKD therefore provides a critical foundation for early intervention, individualized treatment selection, and improved translation of evidence into clinical practice.

Primary prevention of DKD

Primary prevention strategies for DKD differ modestly between diabetes subtypes. In T1D, early and sustained glycemic control remains the most effective preventive strategy, with long-term legacy benefits demonstrated in landmark cohort follow-up studies. In contrast, type 2 diabetes (T2D) requires a broader, multifactorial preventive approach that integrates weight management, blood pressure control, and early initiation of disease-modifying pharmacotherapy in addition to glycemic optimization.

**Figure 3:**
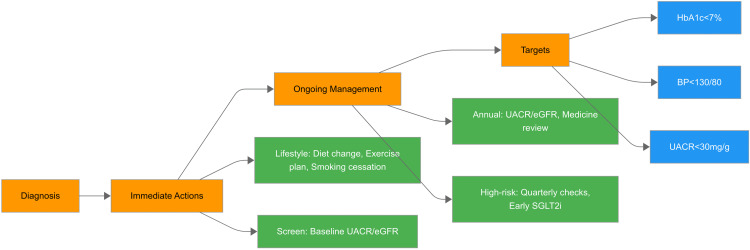
Primary prevention framework for diabetic kidney disease (DKD). The figure illustrates a stepwise approach to the primary prevention of DKD beginning at the stage of diabetes diagnosis. Immediate actions include baseline screening of urine albumin-to-creatinine ratio (UACR) and estimated glomerular filtration rate (eGFR), alongside implementation of lifestyle modifications, such as dietary optimization, regular exercise, and smoking cessation. Ongoing management focuses on maintaining glycemic and blood pressure control and periodic monitoring of renal function. Annual UACR/eGFR assessments and medication reviews are recommended for all patients, with quarterly monitoring and early initiation of sodium-glucose co-transporter 2 (SGLT2) inhibitors for high-risk individuals. The primary prevention targets include achieving HbA1c <7%, blood pressure <130/80 mmHg, and UACR <30 mg/g to prevent or delay the onset of DKD. Image Credit: Shweta Menon

Prevention of DKD necessitates a comprehensive, multidisciplinary strategy, with lifestyle modification forming its foundation (Figure 3). Among dietary interventions, adherence to the Mediterranean and DASH (dietary approaches to stop hypertension) diets has demonstrated renoprotective effects that extend beyond improvements in glycemic control, body weight, and blood pressure. These dietary patterns are associated with reduced albuminuria and slower renal function decline and appear superior to standard dietary approaches in observational and interventional studies [18,19].

Beyond improvements in metabolic and cardiovascular risk profiles, lifestyle interventions have been associated with slower progression of albuminuria, attenuation of eGFR decline, and reduced long-term risk of chronic kidney disease progression, particularly when implemented early in the disease course. Although much of this evidence derives from observational studies and post hoc analyses, the consistency of findings across populations provides a strong biological and clinical rationale for lifestyle modification as a cornerstone of primary DKD prevention.

Historically, low-protein diets were recommended for individuals with chronic kidney disease and reduced glomerular filtration rate (GFR <60 mL/min). However, contemporary evidence supports a moderate protein intake of approximately 0.8 g/kg/day, unless advanced renal dysfunction is present [20]. Excessive protein restriction, particularly in older adults, may increase the risk of malnutrition without providing additional renoprotective benefit [20].

Regular physical activity remains a key determinant of renal health in patients with diabetes. Exercise improves glycemic control, blood pressure, insulin sensitivity, and lipid profiles, while also exerting anti-inflammatory and nephroprotective effects [21]. A meta-analysis demonstrated that structured physical activity is associated with improvements in GFR, reductions in UACR, and a lower incidence of acute kidney injury and kidney failure [22]. Furthermore, higher physical activity levels have been inversely associated with adverse renal outcomes in both diabetic and non-diabetic populations [23].

Tobacco use is a well-established risk factor for vascular injury, accelerating endothelial dysfunction and oxidative stress within renal tissues. Observational studies have consistently demonstrated an association between smoking and more rapid progression of DKD, with diabetic individuals who smoke exhibiting substantially higher rates of progression to ESKD compared with non-smokers, even after adjustment for major clinical covariates [24]. However, these estimates are derived largely from cohort studies and may be influenced by residual confounding related to glycemic control, blood pressure, socioeconomic factors, and treatment adherence.

Although smoking cessation has been robustly associated with reductions in cardiovascular morbidity and mortality [25], direct evidence demonstrating sustained kidney-specific benefits, particularly reduced progression to ESKD, remains limited. Nonetheless, given the strong biological plausibility, consistent epidemiologic associations, and clear cardiovascular benefit, smoking cessation remains a critical component of comprehensive DKD prevention strategies.

Screening Recommendations

Screening strategies for DKD should be stratified according to patient risk profile. Standard-risk individuals include patients with well-controlled diabetes and no additional cardiovascular comorbidities, whereas high-risk patients include those with long-standing diabetes, hypertension, established cardiovascular disease, obesity, or prior evidence of albuminuria. This stratification allows tailoring of screening intensity while optimizing resource utilization.

Routine annual screening for DKD using UACR and eGFR is recommended for all patients with T2D and for individuals with T1D of more than five years' duration [26]. Approximately 25% of patients with T1D develop evidence of kidney damage within 10 years of diagnosis, rising to nearly 50% by 20 years [27]. Targeted screening of at-risk populations using UACR and eGFR has been shown in multiple health-economic and population-based modeling studies to be potentially cost-effective in selected low- and middle-income settings, particularly when integrated with adequate laboratory capacity, follow-up systems, and access to renoprotective therapies, thereby enabling earlier detection and delayed disease progression [28]. However, in settings where diagnostic capacity, treatment availability, or continuity of care is limited, expanded screening alone may not translate into improved renal outcomes and could place additional strain on already constrained health systems. Within this context, prioritizing intensified monitoring for individuals at the highest risk, such as those with hypertension or established cardiovascular disease, may offer the greatest clinical and economic value, with quarterly eGFR assessment and prompt intervention when UACR exceeds 30 mg/g being recommended [29].

Interpretation of urine albumin-to-creatinine ratio results requires recognition of biological variability and potential false-positive elevations, which may occur due to transient factors, such as exercise, acute illness, febrile states, urinary tract infection, or poor glycemic control. Accordingly, confirmation with repeat testing is recommended before establishing a diagnosis of persistent albuminuria. From an implementation perspective, effective DKD screening requires integration into routine diabetes care pathways, with automated reminders, standardized laboratory reporting, and clear follow-up protocols. Risk-stratified screening intervals, confirmation of abnormal results, and linkage to timely therapeutic intervention are essential to translate early detection into meaningful renal risk reduction.

Pharmacologic Interventions

Optimal glycemic control remains a foundational component of primary prevention of DKD. Metformin continues to be recommended as first-line glucose-lowering therapy in the absence of contraindications, given its favorable metabolic profile and long-standing safety record [29]. However, its renoprotective effects are largely indirect and mediated through improved glycemic control rather than kidney-specific mechanisms.

In contrast, SGLT2 inhibitors have demonstrated benefits that extend beyond glucose lowering. Landmark trials, including EMPA-KIDNEY and DAPA-CKD, have shown that SGLT2 inhibitors reduce the risk of chronic kidney disease progression across a broad spectrum of patients with diabetes, including those with preserved or reduced eGFR and varying degrees of albuminuria [30]. These benefits are attributed to renal-specific mechanisms such as reduction of intraglomerular pressure, mitigation of hyperfiltration, and attenuation of inflammatory and fibrotic pathways. While guideline bodies, including the American College of Physicians, strongly endorse their use in appropriate patients with diabetes, reported effect sizes - such as relative risk reductions of up to approximately 30-35% for kidney disease progression - should be interpreted in the context of baseline risk and absolute event rates [31].

From a safety perspective, SGLT2 inhibitors are generally well tolerated, but their use requires consideration of contraindications and precautions, including advanced volume depletion, recurrent genital infections, and risk of euglycemic ketoacidosis in susceptible individuals. Importantly, these agents are associated with a lower risk of hypoglycemia and hyperkalemia compared with several other antidiabetic therapies [32].

Blood pressure control represents another critical pillar of pharmacologic prevention. The RENAAL study demonstrated that systolic blood pressure above 140 mmHg is associated with a substantially increased risk of progression to ESKD, with each 10 mmHg increase in systolic pressure conferring a 7-11% incremental risk [33]. Angiotensin-converting enzyme inhibitors and angiotensin receptor blockers (ARBs) reduce albuminuria and slow DKD progression through both hemodynamic and antifibrotic effects, with reported relative reductions in proteinuria and long-term renal endpoints that are independent of blood pressure lowering alone [33,34]. The American Diabetes Association (ADA) recommends maintaining glycated hemoglobin (A1c) levels below 7% and blood pressure below 130/80 mmHg as evidence-based targets for reducing the risk of DKD [35]. However, translation of these targets into routine clinical practice is often challenging, particularly in low-resource or fragmented healthcare systems, due to socioeconomic constraints, therapeutic inertia, limited access to multidisciplinary care, and inconsistent follow-up. Moreover, intensive glycemic control may not be appropriate for all patients, especially older adults or individuals with advanced comorbidities, in whom individualized targets balancing renal benefit and treatment-related risk are recommended. Addressing these gaps requires not only guideline dissemination and patient education, but also system-level interventions, including integrated care models, provider training, and policy support for structured screening and longitudinal follow-up.

Secondary prevention of DKD

Secondary prevention of DKD focuses on slowing renal function decline and preventing progression to ESKD in patients with established kidney involvement, such as microalbuminuria or mild-to-moderate reductions in eGFR [36]. This stage represents a critical therapeutic window during which timely, combination-based interventions can substantially modify long-term renal and cardiovascular outcomes.

Glycemic Optimization

**Figure 4:**
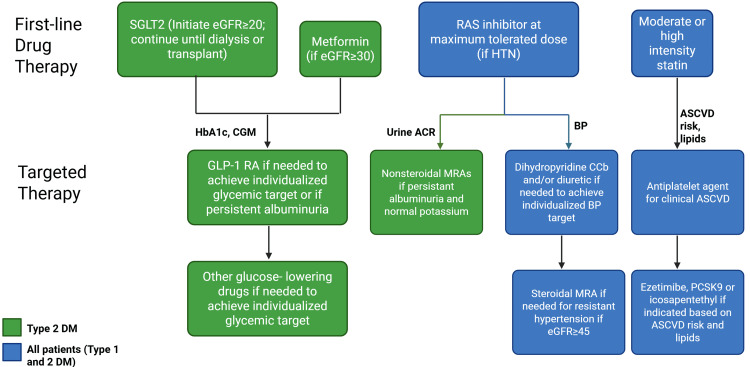
Secondary prevention and guideline-directed medical therapy for diabetic kidney disease (DKD). The figure outlines a comprehensive framework for secondary prevention in patients with established DKD, emphasizing evidence-based pharmacologic interventions. Sodium-glucose co-transporter 2 (SGLT2) inhibitors are recommended for initiation when estimated glomerular filtration rate (eGFR) ≥20 mL/min/1.73 m² and continued until dialysis or transplant. Metformin may be used when eGFR ≥30 mL/min/1.73 m². Glucagon-like peptide-1 receptor agonists (GLP-1 RAs) and other glucose-lowering agents can be added to achieve individualized glycemic targets or when persistent albuminuria exists. For blood pressure control, renin-angiotensin system (RAS) inhibitors at the maximum tolerated dose form the cornerstone, with dihydropyridine calcium channel blockers (CCBs), diuretics, and mineralocorticoid receptor antagonists (MRAs) added as needed for resistant hypertension. Lipid management includes moderate- or high-intensity statins, with adjunctive therapies such as ezetimibe, PCSK9 inhibitors, or icosapent ethyl based on atherosclerotic cardiovascular disease (ASCVD) risk and lipid profile. Antiplatelet agents are indicated for patients with established ASCVD. Image Credit: Mariyam Khan

Hyperglycemia is a central driver of DKD progression, promoting glomerular hyperfiltration, oxidative stress, and cumulative nephron injury. Achieving and maintaining appropriate glycemic control reduces the risk of microvascular complications, including progression of albuminuria (Figure 4). Insulin therapy remains essential in patients with long-standing diabetes and declining β-cell function, particularly when oral agents are insufficient [37]. The ORIGIN trial demonstrated neutral renal outcomes with early basal insulin glargine use while confirming its long-term safety in high-risk individuals [38]. Newer ultra-long-acting insulin analogues, such as degludec, reduce hypoglycemia risk and may be advantageous in patients with chronic kidney disease due to impaired insulin clearance.

Beyond insulin, GLP-1 RAs provide additional benefit. In the LEADER trial, liraglutide reduced new-onset persistent macroalbuminuria by 26% [39], while the SUSTAIN-6 trial demonstrated a 36% reduction in new or worsening nephropathy with semaglutide (HR: 0.64; 95% CI: 0.46-0.88) [40]. These effects appear to be mediated largely through reductions in albuminuria and systemic inflammation rather than direct structural renal remodeling.

Blood Pressure Targets and Optimization

Blood pressure control is vital to secondary DKD prevention, mitigating glomerular capillary hypertension and ischemic injury. The 2017 ACC/AHA guidelines recommend a target blood pressure of <130/80 mmHg for patients with diabetes and chronic kidney disease (CKD) [41]. The ACCORD-BP trial showed no significant reduction in major renal outcomes with intensive systolic blood pressure targeting (<120 mmHg) compared with standard control, although reductions in microalbuminuria were observed [42]. More recently, the 2021 Kidney Disease: Improving Global Outcomes (KDIGO) guidelines - drawing in part from SPRINT trial data - recommend a systolic blood pressure target of <120 mmHg in selected high-risk CKD patients, emphasizing cardiovascular and mortality benefits rather than kidney-specific endpoints [43]. In clinical practice, blood pressure targets should therefore be individualized, balancing potential benefit against risks such as orthostatic hypotension and reduced renal perfusion.

Renin-Angiotensin-Aldosterone System (RAAS) Inhibition

Activation of the RAAS is a key pathogenic mechanism in DKD, contributing to glomerular hypertension, inflammation, and fibrosis. Angiotensin-converting enzyme inhibitors (ACE inhibitors) and ARBs remain first-line therapies for patients with diabetes and albuminuria. The RENAAL trial demonstrated that losartan reduced the risk of doubling of serum creatinine by 25%, ESKD by 28%, and proteinuria by 35% in patients with type 2 diabetic nephropathy (p<0.01 for all) [44]. Similarly, the IDNT trial showed that irbesartan reduced the composite endpoint of doubling of serum creatinine, ESKD, or death by 20% compared with amlodipine [34]. Dual ACE inhibitor and ARB therapy is not recommended due to increased risks of hyperkalemia and acute kidney injury.

The non-steroidal MRA finerenone represents an important adjunctive therapy in this setting. In the FIDELIO-DKD trial, finerenone reduced the risk of kidney failure, sustained ≥40% eGFR decline, or renal death by 18% compared with placebo [3]. The FIGARO-DKD trial, which enrolled patients with earlier-stage DKD, demonstrated a 13% reduction in major cardiovascular events, supporting its role in broader diabetic populations with residual risk despite optimized RAAS blockade [45].

Although landmark trials such as RENAAL and IDNT established RAAS blockade as a cornerstone of therapy in proteinuric DKD, their findings should be interpreted within the context of contemporary clinical practice. These trials predominantly enrolled patients with overt albuminuria, preserved potassium homeostasis, and limited multimorbidity, whereas real-world DKD populations are often older, more comorbid, and at higher risk of hyperkalemia and acute kidney injury. Moreover, these studies predated the widespread availability of SGLT2 inhibitors and nonsteroidal MRAs, raising uncertainty regarding the incremental benefit and optimal sequencing of RAAS inhibition within modern combination regimens. Accordingly, while RAAS blockade remains a foundational component of secondary prevention, it should be individualized rather than applied uniformly or indefinitely, with careful biochemical monitoring and periodic reassessment of renal function, tolerability, and integration with adjunctive renoprotective therapies.

SGLT2 Inhibitors

SGLT2 inhibitors have emerged as foundational agents in secondary DKD prevention, with benefits extending beyond glucose lowering. These agents reduce intraglomerular pressure, attenuate hyperfiltration, and exert anti-inflammatory and antifibrotic effects. In the EMPA-REG OUTCOME trial, empagliflozin reduced the progression of kidney disease by 39%, primarily through reductions in albuminuria and worsening nephropathy [46]. The DAPA-CKD trial demonstrated that dapagliflozin reduced the composite outcome of ≥50% eGFR decline, ESKD, or renal/cardiovascular death by 39%, including in patients without diabetes [47]. Similarly, the EMPA-KIDNEY trial reported a 28% reduction in kidney disease progression or cardiovascular death across a broad CKD population [48]. These findings support early incorporation of SGLT2 inhibitors once albuminuria or eGFR decline is identified, provided no contraindications exist.

Despite consistent evidence of reno- and cardioprotective benefits across a broad spectrum of estimated glomerular filtration rate and albuminuria levels, early initiation of SGLT2 inhibitors remains suboptimal in routine clinical practice, particularly among patients with preserved renal function or minimal albuminuria [46]. While contemporary guidelines increasingly endorse their use beyond glucose lowering, real-world implementation is frequently delayed due to concerns regarding cost, safety (including genital infections and volume depletion), clinician inertia, and limited access within fragmented or resource-constrained healthcare systems. Addressing these barriers will require system-level strategies, including clinician education, simplified prescribing pathways, reimbursement support, and integration of SGLT2 inhibitors into early DKD care algorithms rather than reserving them for advanced disease stages.

Integrative Clinical Perspective

In secondary prevention, therapy sequencing and combination are critical. RAAS blockade and SGLT2 inhibitors form the therapeutic foundation, with GLP-1 receptor agonists and finerenone added based on residual metabolic, albuminuric, or cardiovascular risk. Translation of trial evidence into routine practice requires careful patient selection, monitoring for adverse effects, and consideration of real-world factors such as tolerability, comorbidity burden, and access to care. A structured, stepwise approach to combination therapy offers the greatest potential to slow DKD progression while minimizing treatment-related harm.

Comparative effectiveness of guideline-directed therapies

Established Guideline-Directed Therapies

RAAS inhibition remains the historical foundation of renoprotective therapy in DKD. However, RAAS blockade alone is insufficient to halt disease progression, prompting the development and evaluation of adjunctive disease-modifying therapies. Among these, SGLT2 inhibitors have emerged as the most robustly validated add-on therapy, with consistent renal and cardiovascular benefits demonstrated across large randomized controlled trials [49].

The CREDENCE trial demonstrated that canagliflozin significantly reduced the risk of ESKD, doubling of serum creatinine, and renal or cardiovascular death by approximately 30% in patients with type 2 diabetes and albuminuric CKD already receiving RAAS blockade [50]. Similarly, the DAPA-CKD trial confirmed that dapagliflozin reduced major kidney outcomes across a broad CKD population, including patients without diabetes [51]. Collectively, these trials established RAAS inhibition plus SGLT2 inhibition as the contemporary therapeutic backbone for secondary prevention of DKD (Table 1).

**Table 1:** Characteristics of various kidney outcome studies. a = End-stage kidney disease, doubling of the serum creatinine from baseline sustained for at least 30 days, or death from renal or CV disease. b = First occurrence of any of the following: a decline of at least 50% in the eGFR, onset of ESKD, or death from renal or cardiovascular causes. c = Incident macroalbuminuria, plus an increase in the urinary albumin-to-creatinine ratio of ≥30% from baseline, a sustained decrease in the eGFR of ≥40% for ≥30 days, renal-replacement therapy for ≥90 days, or a sustained eGFR of <15 mL per minute per 1.73 m2 for ≥30 days. T2D - type 2 diabetes; GFR - glomerular filtration rate; eGFR - estimated glomerular filtration rate; HbA1C - hemoglobin A1C; RAAS - renin-angiotensin-aldosterone system; SGLT2 - sodium-glucose cotransporter 2; ACR - albumin-to-creatinine ratio; UACR - urine albumin-creatinine ratio; MRA - mineralocorticoid receptor antagonism; CV - cardiovascular; ASCVD - atherosclerotic cardiovascular disease; DDP-4 -dipeptidyl peptidase 4

Study	Study Drug	Population included	Statistical Value (p value)	Limitations of the Study	
DAPA-CKD [45]	Dapagliflozin vs. placebo	Adults with or without T2D with eGFR 25-75 mL/min/1.73 m^2^, UACR 200-5000 mg/g	P<0.001 (for primary composite outcome^b^)	Patients with type 1 diabetes and participants who had received immunotherapy for kidney disease in the last 6 months were excluded. Reversal of initial dip in GFR after discontinuation of the drug was not assessed as eGFR values were not collected after the trial	
CREDENCE [48]	Canagliflozin vs. placebo	T2D patients with HbA1C of 6.5-12%, eGFR 30-<90 mL/min/1.73 m^2^, UACR >300-5000 mg/g	P=0.00001 (for primary composite outcome^a^)	Patients with very advanced kidney disease, nonalbuminuric or microalbuminuric disease, and kidney disease believed to be due to conditions other than T2D were excluded. Power of some secondary outcomes could have been limited due to early stoppage of the trial	
SGRASS-DKD [49]	RAAS inhibitor + SGLT2 inhibitor	Patients with type 1 or 2 diabetes with urinary spot ACR >30 mg/g and/or eGFR less than 60 mL/min/1.73 m^2^	P<0.001 (for glycemic measures and UACR)	Patients with eGFR <30ml/min/1.73m^2 ^were excluded. Small sample size and short duration of study (1 year)	
	Eplerenone vs. placebo	Patients with diagnosis of T2D with UACR >300 mg/g, eGFR 30-90 mL/min/1.73 m^2^	P<0.05 (significant increase in Ang I and Ang-(1-7))	The study is of exploratory design hence its findings cannot be generalized. Only 3 were women, preventing the study from investigating the impact of gender. There is no long-term follow-up of patients	
Effect of MRA and ACE inhibition [50]					
AMPLITUDE-O [52]	Efpeglenatide at a dose of 4 mg or 6 mg or placebo.	T2D patients with HbA1C >7%, at least 18yrs of age with history of CV disease or 50 yrs (male)/55 yrs (female) with eGFR 25.0 to 59.9 mL/min/1.73 m^2^ and at least one CV risk factor	P<0.001 ( for renal composite outcome event^c^)	Short follow-up period. Selection for already existing CV or kidney disease limits the power of trial and its generalizability to lower risk persons with T2D	
Post hoc analysis from the DECLARE-TIMI 58 [53]	Dapagliflozin vs. placebo	T2D patients with either established atherosclerotic CV disease or multiple risk factors for ASCVD, HbA1C 6.5-12%, creatinine clearance equal to or more than 60 mL/min	P<0.0001 (for fast and severe eGFR decline)	Measurement of UACR and creatinine was infrequent since it was a CV outcome trial and not a kidney outcome trial. This made understanding of any acute changes in kidney more difficult	
Retrospective study [54]	DDP-4 inhibitors	T2D patients treated with anti-hyperglycemic agents	P<0.001 (for eGFR decline from baseline by equal to or more than 30%)	Patients with history of established renal failure or kidney transplantation were excluded. Short investigation period to reflect long-term renoprotective effects of DDP-4 inhibitors	

Adjunctive and Emerging Therapies

Persistent aldosterone signaling (“aldosterone escape”) despite RAAS blockade has renewed interest in MRAs. Early exploratory studies with eplerenone suggested potential renoprotective effects when combined with ACE inhibition, mediated in part through modulation of angiotensin-(1-7) signaling; however, these findings derive from small, short-duration studies and should not be generalized [50].

In contrast, finerenone, a non-steroidal MRA, has demonstrated clinically meaningful benefits in large outcome trials. The FIDELIO-DKD and FIGARO-DKD programs established finerenone as an effective adjunct to RAAS blockade in reducing renal and cardiovascular outcomes in appropriately selected patients. The ongoing CONFIDENCE trial, which is evaluating finerenone in combination with SGLT2 inhibitors, remains investigational and not yet practice-changing [53].

The AMPLITUDE-O trial demonstrated that efpeglenatide reduced kidney disease progression and need for renal replacement therapy, irrespective of background SGLT2 inhibitor use [54]. However, renal endpoints were secondary outcomes, and benefits appear largely mediated through albuminuria reduction and cardiovascular risk modification, rather than direct structural renal protection.

Post-hoc analyses from cardiovascular outcome trials, such as DECLARE-TIMI 58, suggest additive renal benefits of dapagliflozin independent of GLP-1 RA therapy [55]. While hypothesis-generating, such findings should be interpreted cautiously due to infrequent renal outcome ascertainment and post-hoc design.

Evidence supporting DPP-4 inhibitors remains limited. Retrospective analyses suggest modest associations with slower eGFR decline [56], but no large randomized kidney outcome trials support their use as renoprotective agents, and they should not be considered disease-modifying therapies in DKD.

Endothelin receptor antagonists (ERAs) have demonstrated reductions in albuminuria in randomized trials [57], but concerns regarding fluid retention and heart failure have constrained their clinical adoption. Their role remains investigational pending safer formulations or combination strategies.

Several experimental approaches, including cell-based therapies, JAK-STAT pathway inhibition (e.g., baricitinib), and Nrf2 activation (e.g., bardoxolone methyl), have shown promising biomarker or short-term renal effects [58-60]. However, these studies are small, exploratory, and limited by short follow-up and safety concerns. None currently warrants incorporation into routine clinical practice.

Clinical Perspective

Taken together, comparative effectiveness data support a tiered therapeutic hierarchy in DKD. RAAS blockade and SGLT2 inhibitors represent established, practice-changing therapies, while finerenone and GLP-1 receptor agonists serve as evidence-supported adjuncts in selected patients. In contrast, DPP-4 inhibitors, ERAs, and novel molecular or cellular therapies should be regarded as investigational, pending definitive outcome trials. Persistent barriers, including clinical inertia, safety concerns, cost, and limited access, continue to hinder optimal implementation of guideline-directed therapy, underscoring the need for system-level strategies to translate evidence into routine care.

Current clinical guidelines for DKD management

Contemporary clinical guidelines for DKD increasingly emphasize early risk stratification, combination therapy, and integrated cardio-renal protection, reflecting the expanding evidence base for disease-modifying agents. Although developed by different professional societies, major guidelines demonstrate substantial consensus on foundational principles, with divergence primarily in treatment thresholds, sequencing, and strength of recommendations.

Areas of Consensus

Across the ADA, KDIGO, European Society of Cardiology (ESC), and American Heart Association (AHA) guidelines, there is a strong consensus that routine screening with estimated glomerular filtration rate and urine albumin-to-creatinine ratio is essential for early detection of DKD. All guidelines consistently recommend RAAS blockade for patients with albuminuria and identify SGLT2 inhibitors as foundational therapies for reducing renal and cardiovascular risk in DKD. Blood pressure control and statin therapy remain central components of risk modification strategies, while glycemic targets are uniformly emphasized to be individualized based on patient comorbidity burden, hypoglycemia risk, and overall clinical context.

These recommendations are generally supported by high-quality randomized controlled trial evidence and are consistently classified as strong recommendations across guidelines (Table 2).

**Table 2:** The latest clinical guidelines for the management of diabetic kidney disease (DKD). AHA = American Heart Association; ADA = American Diabetes Association; ESC = European Society of Cardiology; JNC = Joint National Committee; KDIGO = Kidney Disease: Improving Global Outcomes

Clinical Guideline	Target Population	Glycemic Control	Blood Pressure Management	Cardiovascular Management
KDIGO (2024) [29]	Patients with diabetes and CKD (G1–G5)	• Individualized HbA1c <6.5–8.0% (non-dialysis) • CGM/SMBG recommended • Metformin if eGFR >30 • GLP-1 RA only if SGLT2i not tolerated/inadequate	• Target SBP <120 mmHg (if tolerated) • ACEi/ARB first-line if UACR ≥30 mg/g • Avoid dual RAAS blockade • Sodium <2 g/day; ≥150 min/week physical activity	• Statin or statin–ezetimibe for age >50 • First-line SGLT2i when eGFR ≥20; GLP-1 RA if not tolerated • Aspirin for secondary prevention • Smoking cessation, exercise
AHA (2022) [41]	Adults with cardiovascular risk including T2DM	• No specific HbA1c target • Metformin first-line • Add SGLT2i or GLP-1 RA if ASCVD risk persists	• BP <130/80 mmHg for CKD/diabetes • ACEi/ARB/CCB as first-line	• Moderate/high-intensity statins based on ASCVD risk • Aspirin for secondary prevention • SGLT2i in CKD; GLP-1 RA in ASCVD
ADA Standards of Care (2025) [61, 62]	Adults with diabetes, especially T2DM	• HbA1c <7% for most; individualized 6.5–8% • Metformin first-line if eGFR ≥30 • GLP-1 RA if SGLT2i not appropriate	• <140/90 mmHg for most • <130/80 mmHg if high CV risk or UACR ≥30 mg/g • ACEi/ARB if albuminuria present • Thiazides/CCBs/β-blockers as add-on	• Moderate/high-intensity statins 40–75 yrs with DKD/CVD risk • Strong recommendation for SGLT2i/GLP-1 RA with CV benefit • Aspirin for secondary prevention; selective use in primary
ESC (2023) [63, 64]	Patients with CVD, diabetes, and/or CKD	• HbA1c 6.5–8%; preferably <7% • SGLT2i + GLP-1 RA first-line in T2DM with CKD • Metformin as add-on	• <130 mmHg in age <65 (avoid <120) • 130–139 mmHg in age >65 • RAAS blockade preferred if albuminuria • RAAS + CCB/thiazide for early BP control	• Statins first-line • LDL targets: <100 mg/dL (moderate risk), <55 mg/dL (very high risk) • SGLT2i first-line; GLP-1 RA if intolerant • Antiplatelet only for secondary prevention
JNC 8 [65]	• Adults ≥18 years with hypertension • Includes diabetics and adults with CKD	• No glycemic targets specified (focus is hypertension)	• Treat ≥60 yrs to goal <150/90 mmHg (strong evidence) • Treat ages 30–59 yrs to DBP <90 mmHg (strong evidence) • Insufficient evidence for SBP goal in <60 yrs → expert opinion recommends <140/90 mmHg • Same BP goals for adults with diabetes or CKD as general hypertensive population • First-line agents for non-Black adults (incl. diabetes): ACEi, ARB, CCB, or thiazide-type diuretic • First-line for Black adults (incl. diabetes): CCB or thiazide-type diuretic • CKD (any race): initiate ACEi or ARB to improve kidney outcomes (moderate evidence)	• Not specifically addressed beyond BP reduction for cardiovascular risk management

Key Areas of Divergence

Despite broad alignment, important differences exist. The ADA Standards of Care (2025) continue to recommend an HbA1c target of <7% for most patients, with individualization (6.5-8.0%) for selected populations, and maintain a blood pressure target of <130/80 mmHg when safely achievable [61,62]. In contrast, the ESC 2023 guidelines adopt a wider HbA1c range (6.5-8.0%) and prioritize combined SGLT2 inhibitor and GLP-1 receptor agonist therapy earlier in patients with type 2 diabetes and chronic kidney disease [63,64].

The KDIGO 2024 update further broadens SGLT2 inhibitor recommendations to patients with proteinuria irrespective of diabetes status and emphasizes systolic blood pressure targets <120 mmHg when tolerated, reflecting cardiovascular outcome trial data rather than kidney-specific endpoints alone [65]. These recommendations, while evidence-informed, are often classified as conditional, acknowledging variability in patient tolerance and healthcare infrastructure.

Older guidelines, such as JNC 8, although historically influential, reflect earlier evidence and recommend less intensive blood pressure targets, underscoring the evolution of DKD management toward more aggressive risk reduction [66].

Barriers to Guideline Implementation

Despite strong guideline alignment, implementation gaps remain pervasive. Patient-level barriers include suboptimal adherence, limited disease awareness, and concerns regarding polypharmacy and adverse effects [67-69]. Provider-level challenges include time constraints, limited familiarity with rapidly evolving evidence, and therapeutic inertia, even in the presence of strong recommendations [70,71]. At the system level, fragmented care pathways, limited health IT infrastructure, and financial barriers restrict access to disease-modifying therapies such as SGLT2 inhibitors, GLP-1 receptor agonists, and non-steroidal MRAs [72-74].

In routine practice, RAAS blockade and SGLT2 inhibitors represent the strongest, most consistently endorsed interventions across guidelines, with GLP-1 receptor agonists and finerenone serving as adjunctive therapies based on residual risk and patient characteristics. Blood pressure and glycemic targets should be individualized, particularly in older or multimorbid patients. Effective DKD management requires not only guideline adherence but also system-level support, including coordinated care models, access to affordable therapies, and structured follow-up [75-78].

Future directions and research gaps

Despite substantial advances in the management of DKD, several high-impact research gaps remain that must be addressed to further improve patient outcomes. Future progress will depend less on expanding the number of available therapies and more on optimizing patient selection, timing of intervention, and integration of emerging tools into routine care. Key priorities can be broadly divided into near-term clinical opportunities and longer-term investigational approaches.

Near-Term Clinical Priorities

One of the most pressing gaps lies in refining the renal-specific role of newer glucose-lowering therapies beyond their established metabolic and cardiovascular benefits. While agents such as GLP-1 RAs have demonstrated reductions in albuminuria and favorable cardiovascular outcomes, their long-term impact on hard renal endpoints, particularly in advanced chronic kidney disease, remains incompletely defined [79-81]. Future trials should focus on kidney-specific outcomes, durability of benefit, and safety in patients with severe renal impairment rather than surrogate markers alone.

Similarly, although certain adjunctive therapies have demonstrated acceptable safety profiles in early-stage CKD, their incremental renoprotective value beyond existing guideline-directed therapy remains uncertain, underscoring the need for pragmatic trials that evaluate additive benefit in contemporary multidrug regimens rather than isolated efficacy [82,83].

Longer-Term and Experimental Directions

Beyond pharmacotherapy, personalized medicine represents a major frontier in DKD care. The marked heterogeneity in disease progression limits the effectiveness of uniform treatment algorithms, highlighting the need for tools that enable early risk stratification and individualized therapy [84]. Circulating biomarkers such as tumor necrosis factor receptors 1 and 2 have shown promise in identifying patients at high risk of progression to ESKD, even in the absence of overt albuminuria [85]. Additional markers, including kidney injury molecule-1 and interleukin-18, may further refine prognostication by capturing tubular injury and inflammatory activity [86].

However, the clinical utility of biomarkers remains limited by a lack of standardization, cost considerations, and uncertainty regarding how biomarker-guided strategies should alter management decisions. Future research should therefore prioritize validating biomarker panels that meaningfully inform treatment initiation, intensification, or monitoring, rather than focusing solely on predictive associations.

Other experimental strategies, including novel anti-inflammatory pathways, endothelin receptor modulation, and advanced biologic or cell-based therapies, remain early in development and should be considered investigational until supported by adequately powered outcome trials demonstrating safety and clinically meaningful benefit.

In summary, the most impactful future advances in DKD are likely to arise from earlier identification of high-risk patients, improved targeting of existing therapies, and integration of biomarker-driven risk stratification, rather than from expansion of therapeutic classes alone. Bridging the gap between mechanistic discovery and pragmatic clinical application remains the central challenge for future DKD research.

## Conclusions

DKD remains a major global health burden, driven by rising diabetes prevalence and persistent gaps in early detection and effective treatment. Advances in understanding the metabolic, hemodynamic, and inflammatory mechanisms of DKD have reinforced the importance of early screening, lifestyle intervention, and optimized control of glycemia, blood pressure, and albuminuria. Contemporary guideline-directed medical therapy, including RAAS blockade, SGLT2 inhibitors, GLP-1 receptor agonists, and non-steroidal mineralocorticoid receptor antagonists, now provides a robust, evidence-based framework to reduce both renal and cardiovascular risk.

However, the clinical impact of these therapies remains limited by suboptimal real-world uptake, driven by therapeutic inertia, access barriers, and fragmented care delivery. Improving outcomes will require patient-centered, systems-based approaches, including coordinated multidisciplinary care, clinician education, structured screening and follow-up pathways, and equitable access to disease-modifying therapies. Ultimately, consistent and timely implementation of guideline-directed therapy-tailored to individual patient risk and context-offers the greatest opportunity to slow DKD progression and reduce its long-term renal and cardiovascular consequences.
